# Considerations for the Use of Stereotactic Radiosurgery to Treat Large Arteriovenous Malformations

**DOI:** 10.3390/biomedicines12092003

**Published:** 2024-09-03

**Authors:** Jong Hyun Lim, Myung Ji Kim

**Affiliations:** Department of Neurosurgery, Korea University Ansan Hospital, Korea University College of Medicine, 123 Jeokgeum-ro, Danwon-gu, Ansan 15355, Gyeonggi-do, Republic of Korea; phoenix118@naver.com

**Keywords:** arteriovenous malformation (AVM), stereotactic radiosurgery (SRS), gamma knife radiosurgery, adverse radiation effect (ARE), radiation-induced change (RIC), volume-staged, dose-staged, obliteration, intracranial hemorrhage, large-volume

## Abstract

Stereotactic radiosurgery (SRS) is an effective treatment strategy for cerebral arteriovenous malformations (AVMs). Aggressive treatment achieving complete obliteration is necessary to prevent further intracranial hemorrhage and neurological deficits. However, SRS treatment of large AVMs (>10 cm^3^) is challenging. To prevent toxicity in the normal brain tissue, it is imperative to reduce the radiation dose as the lesion volume increases; however, this also reduces the rate of obliteration. In this study, we review the various radiosurgical approaches for treating large AVMs and their outcomes, and suggest ways to improve treatment outcomes during SRS for large AVMs.

## 1. Introduction

Stereotactic radiosurgery (SRS) is an effective treatment strategy for cerebral arteriovenous malformations (AVMs), particularly small-to medium-sized AVMs [1,2]. Radiation causes endothelial and subendothelial damage, which leads to cellular proliferation, extracellular matrix expansion, and eventually, total obliteration of the vessel wall structure [3]. Aggressive treatment is necessary to achieve complete obliteration in order to prevent intracranial hemorrhage, seizures (epilepsy), and focal neurologic deficits due to the steal phenomenon. Complete obliteration of the AVM nidus is accomplished in 70–95% of patients within a period of 3 to 5 years of SRS [4]. However, SRS has certain limitations for AVMs greater than 10 cm^3^ in volume. To prevent toxicity to the normal tissues, it is necessary to reduce the radiation dose as the lesion volume increases, thus reducing the rate of obliteration. In this study, we reviewed the existing literature on managing large AVMs, and suggested ways to improve treatment outcomes during SRS for large AVMs.

## 2. Endovascular Embolization

Endovascular embolization, SRS, and surgical resection, alone or in combination, are all effective treatment options for AVMs. Endovascular embolization and SRS are commonly used together for larger and more complex AVMs, particularly those that have ruptured [5]. Using embolization as a neoadjuvant treatment prior to SRS has several benefits, including lowering the size of AVMs, enabling the delivery of a higher radiation dose to a smaller target area, and leading to a higher rate of complete obliteration and fewer complications [2], as well as the elimination of high-risk characteristics, such as AVM-related aneurysms and high-flow shunts [6,7,8], and the alleviation of symptoms related to arterial steal or venous hypertension [9]. Nevertheless, some authors have proposed that embolization prior to SRS has various disadvantages. These include challenges in accurately identifying the AVM during SRS due to the presence of embolic material [10], the creation of collateral feeding vessels [11], the induction of hypoxia (which reduces the responsiveness of the AVM to radiation) [12], and dose attenuation of embolic agents [13,14], which ultimately reduces its obliteration rate. Furthermore, approximately 12–15% of patients have delayed nidus recanalization following either particle or glue embolization [15,16,17]. A multi-institutional retrospective study enrolling 257 patients with large-volume AVMs (median volume, 23.25 cc) who underwent volume-staged SRS (VS-SRS) revealed that prior embolization was associated with poor outcomes [18]. Furthermore, one meta-analysis concluded that the adjuvant use of endovascular embolization to treat associated aneurysms and high-flow fistulas correlated with a lower hemorrhage rate, whereas more aggressive embolization aimed at complete obliteration was associated with a higher rate of periprocedural intracranial hemorrhage [19]. Kim et al. previously suggested that neoadjuvant embolization was a significant negative predictive factor for obliteration 36 months after gamma knife radiosurgery (GKRS) in AVMs with a large volume and complex angioarchitecture in the single-center retrospective study including 228 patients (29 neoadjuvant-embolized, 19 adjuvant-embolized, and 180 nonembolized patients) [20]. This finding is consistent with those of previous matched cohort studies [14] and meta-analyses [21,22]. Hasegawa et al. retrospectively analyzed the treatment outcomes of 1246 patients in a single institution. The study revealed embolization before GKRS was negatively associated with nidus obliteration [23]. However, in a recent case-control study with propensity score matching conducted by the same group, there was no significant difference in either the nidus obliteration rate or cumulative hemorrhage between SRS only and embolization+SRS groups. Most patients in the embolization+SRS group (88%) underwent embolization with *n*-butyl-2-cyanoacrylate [24]. Additionally, a multicenter matched cohort study to compare outcomes of SRS with vs without upfront Onyx embolization revealed that pre-SRS Onyx embolization did not appear to negatively influence outcomes after SRS. The authors proposed that the use of neoadjuvant embolization should be carefully considered for selected large-volume or high-risk AVMs with complex angioarchitecture [25]. Another matched cohort study comprising 17 patients in the embolized group (median volume 17 cc) and 35 patients in the non-embolized group (median volume 13.1 cc) reported that the embolized group had a significantly higher incidence of repeat SRS, and collateral flow and neovascularization were more frequently observed in the embolized non-obliterated AVMs [26]. Although we expected that the potential benefits of embolization would reduce the risk of latent period hemorrhage, particularly recurrent bleeding from previously ruptured AVMs, we suggested considering recanalization following embolization and the adjuvant use of endovascular embolization instead of curative intent.

## 3. Hemorrhage

Intracranial hemorrhage is the most devastating complication of AVMs; if left untreated, the overall risk of spontaneous hemorrhage from a brain AVM varies between 2% and 5% annually [10,27,28]. Moreover, previous studies have demonstrated that the occurrence of an initial AVM rupture significantly increases the risk of subsequent rupture. Prior hemorrhage was identified as a significant contributing factor to subsequent bleeding [29,30,31]. Previous studies have also indicated that smaller AVMs exhibit a higher incidence of hemorrhage than larger AVMs [32,33]. Nevertheless, recent investigations in the field of natural history have indicated that a larger AVM size is correlated with a heightened likelihood of subsequent hemorrhage [34,35]. The annual rate of hemorrhage after radiosurgery for large AVMs varies from 3.3% to 12.41% [36].

In the case of large AVMs, one potential drawback of VS-SRS is that partial AVM irradiation may increase the bleeding risk by redistributing blood flow within the AVM to non-irradiated patients [37,38]. Colombo et al. also found that the hemorrhage risk for partially treated AVMs was greater (7/27 patients, 26%) than that for completely irradiated AVMs (8/153, 5%) [39]. Although some early studies have revealed an increased risk of bleeding after SRS, larger and more detailed analyses have demonstrated that the risk of hemorrhage is either unchanged or decreased following SRS for AVMs [40,41,42,43,44]. Indeed, one previous meta-analysis that included only AVMs with an AVM score greater than 2 reported that the annual hemorrhage rate was 3.22%, the annual hemorrhage rate for unruptured large AVMs was 3.53%, and the annual hemorrhage rate for previously ruptured large AVMs was 6.10% following SRS, which was comparable to the baseline rupture rates reported for untreated AVMs [45].

## 4. Adverse Radiation Effect 

Approximately 30% of patients with AVM show radiation-induced changes (RICs) in the brain on magnetic resonance (MR) T2 scans following SRS. However, these features are asymptomatic in two-thirds of affected individuals. As a result, symptomatic adverse radiation effects (ARE) arise in approximately 9% of patients, while 3% have a permanent deficit [46,47,48,49]. Previous studies have indicated that a sudden alteration in venous drainage could result in substantial T2 changes, as well as the development of severe edema following SRS [50,51], a phenomenon which may be explained by the existence of occlusive hyperemia [52]. This finding supports the notion that hemodynamic changes other than radiation injury may be attributed to ARE [53]. A recent study further suggested that an imbalance between inflow and outflow capacity and a lower vein-artery (VA) ratio are both strong predictors of ARE [53]. Conversely, Kim et al. reinforced the idea that radiation injury to the intervening brain parenchyma within the nidus could explain AREs. They found that a greater proportion of brain tissue between the nidus and 50% isodenseline (IDL) was significantly correlated with RICs [54]. 

A higher incidence of ARE was also observed in patients with large AVMs treated with SRS. Miyawaki et al. previously reported that AVMs with a volume ≥ 14 cc treated with a dose of ≥16 Gy had a 72% frequency of radiation necrosis, while surgical resection was required in 22% of patients [55]. Han et al. previously reported that 33.3% of AVMs with volumes ranging from 4 to 14 cc experienced postradiosurgical changes, with AVMs > 14 cc having 3 times fewer AREs. They ascribed this result to their dosimetry strategy of preventing radiation effects by prescribing a dose of approximately 10 Gy to large AVMs [56].

Administering conventional therapeutic doses of radiation to treat large AVMs is associated with a higher likelihood of AREs in the adjacent brain tissue. While there is a clear correlation between the dose and AVM obliteration, the prescription of the dose must consider the potential risk of ARE [57,58,59,60]. The “12-Gy-volume” refers to the total volume of tissue receiving 12 Gy or more (including the target). Flickinger et al. found that the “12-Gy-volume” reflects the risk of developing postradiosurgery imaging changes, while the location of the AVM significantly influenced whether the postradiosurgery imaging changes were symptomatic or not [48]. They also proposed that it would not be advisable to reduce the chance of obliteration using lower doses solely to avoid temporary and mild postoperative sequelae.

When treating large AVMs, it is necessary to decrease the prescribed dose to minimize the risk of AREs. However, this resulted in a decrease in the complete obliteration rate of the AVMs. Hence, attaining complete obliteration of large AVMs in a single session is challenging. Staged SRS with multiple irradiations has previously been documented. The rationale for the volume-staged SRS (VS-SRS) was proposed by Pollock et al., who compared the radiation dosimetry of VS-SRS with that of hypothetical single-session procedures in 10 patients. VS-SRS resulted in an average reduction of 11.1% in the “12-Gy-volume”, while the “non-AVM 12-Gy-volume” was decreased by an average of 27.2%. This study concluded that performing VS-SRS on large AVMs led to reduced radiation exposure to the surrounding brain tissue [37]. The reported ARE rates of ARE following VS-SRS range from 11.2% to 14% [38,61,62,63,64,65]. A recent retrospective study comparing large AVMs treated with regular-dose (18–22 Gy) and low-dose (<18 Gy) SRS reported that regular-dose SRS significantly contributed to an increase in the obliteration rate and a decrease in significant neurological events and hemorrhage. The authors also proposed that single-session SRS could be acceptable for AVMs with volumes up to 20 cc [66]. 

## 5. Staged SRS

Radiosurgical methods for treating large AVMs include single-session dose-stage (DS-SRS) and volume-stage (VS-SRS) procedures. Typically, a higher cure rate is not achieved when the AVM nidus volume is large, for two reasons. The primary reason is that only partial coverage of the actual nidus is commonly achieved, as there is a worry of causing intolerable damage to the surrounding normal brain tissue. The second reason is the use of a low prescribed marginal dose to minimize the harmful effects of radiation on the treated area [67]. We reviewed two radiosurgical approaches to achieve favorable outcomes in the treatment of large AVMs.

### 5.1. Dose-Staged SRT/SRS (DS-SRS) 

Previous studies described dose staging as either hypofractionated stereotactic radiotherapy (HSRT) or repeat SRS. HSRT involves the delivery of several small doses of radiation to the AVM over a few weeks. The main rationale for this approach is the fractionation effect in the normal brain, i.e., the brain tissue adjacent to the target volume can tolerate a higher total dose. Repeat radiosurgery employs a higher initial dose (less than a single session for small-to-medium-sized AVMs), while additional sessions are performed after several months or years, if there is no evidence of obliteration [64]. 

The nidus of AVMs may have a small α/β ratio, similar to that of late-responding normal tissues. Nevertheless, the real α/β ratios for AVMs, as well as for normal vessels or normal neural structures, remain only poorly understood. Assuming an α/β ratio of 2.0 Gy for normal tissue, which is widely recognized for late effects following radiotherapy, a dose of 25 Gy in 5 fractions and 32 Gy in 4 fractions would have equivalent effects to doses of 12.3 and 17 Gy delivered in a single fraction, respectively [68]. The results of HSRT have revealed a decrease in treatment-related toxicity, while still achieving an obliteration rate of 20–40%, which is lower than that of RS [68,69,70,71,72,73,74]. Unlike conventional HSRT, which divides the dose evenly into several fractions, partial hypofractionation is a method to boost complementary parts of the target volume in different fractions to improve the therapeutic ratio and biological dose reduction of the normal brain in the treatment of large AVMs [75]. 

Repeat SRS involves administering a low dose of radiation to the entire AVM nidus, followed by a waiting period of three to four years, after which the remaining AVM is treated. Kim et al. previously reported an overall obliteration rate of 34.1% in 44 patients who underwent repeat SRS, maintaining an interval of at least 3 years between procedures (mean AVM nidus volume, 48.8 cm^3^, mean marginal dose, 13.9 Gy) [76]. Another study that included 89 patients (median volume, 14 cm^3^; median marginal dose, 15 Gy) documented an estimated obliteration rate of 62% following repeat SRS in the retrospective study. However, the annual incidence of hemorrhage is high (7%), with 35% occurring within the first year of initial treatment [74]. Chytka et al. retrospectively compared the results of staged treatment with single-session radiosurgery for AVM (≥15 cm^3^) and suggested performing repeat radiosurgery if complete obliteration was not accomplished within 3 years of SRS [77]. 

Mantziaris et al. pooled data from 14 multicenter experiences with 505 patients undergoing repeat SRS. This study included 167 AVMs (33.1%) with a Virginia Radiosurgery AVM Scale (VRAS) score of 3 and 68 AVMs (13.5%) with a VRAS score of 4. They reported 59.4% of obliteration rate, 5.6% of post-SRS hemorrhage, and 5.6% of symptomatic RIC. The authors identified that a larger nidus volume and brainstem/basal ganglia involvement were negatively correlated with a favorable outcome following repeat SRS [78]. A recent systemic review and meta-analysis conducted by Shaaban et al. included 32 AVMs with a Spetzler–Martin (SM) grade IV and 6 AVMs with SM grade V. The authors suggested that repeat SRS could be used for SM IV and V AVMs after a period of 3 to 5 years after the initial SRS for incompletely obliterated AVMs in the International Stereotactic Radiosurgery Society Practice Guidelines [79].

### 5.2. Volume-Staged SRS (VS-SRS)

VS-SRS is a technique in which the nidus is divided into multiple volumes and treated in consecutive sessions with 3–6-month intervals between treatments. This method decreases the amount of radiation received by the normal brain, potentially minimizing the risk of toxicity, while ensuring that the prescribed doses are adequate [18,37,61]. This technique is based on the hypothesis that higher radiation doses can decrease the risk of hemorrhage in large AVMs by improving the rate at which they are obliterated. Additionally, it is believed that the normal tissues surrounding AVMs undergo sublethal radiation repair before progressing to the next session [80]. The initial series divided the nidus into two volumes, independent of size, and administered doses of <17 Gy for each stage. VS-SRS led to only a 21% chance of near or complete obliteration at 5 years [62]. The dosage used at each stage was consistently increased. When doses ≥ 17 Gy were used, the likelihood of complete obliteration after 5 years was found to be 68% [62]. In one prospective study by Kano et al., it was found that there was a 62% chance of obliteration when a dose ≥ 17 Gy was administered per volume stage after a period of 5 years [64]. In addition, Seymour et al. proposed in the multi-center retrospective study that increasing the dose beyond 17.5 Gy was strongly associated with an increased rate of partial response, complete obliteration, and cure. Conversely, the cohort that received extensive treatment experienced a higher incidence of latent period hemorrhage than anticipated [18]. In contrast, Franzin et al. identified that radiation doses of up to 20 Gy with a longer time interval between the stages (15 ± 6 months) were safely administered in 20 VS-SRS procedures for large AVMs (median volume 15.9 cm^3^) in the prospective study [65]. Another retrospective study investigating how to improve outcomes in VS-SRS suggested that ≥20-Gy volume coverage was significantly correlated with higher total obliteration rates. Further, results showed that when the margin dose was ≥17 Gy and the 20-Gy SRS volume included ≥ 63% of the total target volume, the obliteration rates increased to 61% at 5 years, and 70% at 10 years. The authors recommended adding additional isocenters with low weights within the volume described by 17 Gy to increase the volume of AVM receiving ≥20 Gy [81]. Regarding the risk of ARE following VS-SRS, 257 patients from nine different radiosurgical centers were retrospectively analyzed (median volume, 23.25 cm^3^ and median marginal dose, 17 Gy). The authors reported that 25% of patients developed ARE, of which 7.4% were permanent. They revealed that the maximal linear dimension of the Z (craniocaudal) dimension significantly correlated with toxicity (threshold length, 3.28 cm), possibly because these large lesions must either be displaced or contain an eloquent cortex, which may lead to symptomatic ARE [82].

Typically, the time interval between sessions was intended to be 3–6 months. Recommendations have been made by other studies to decrease the time interval between treatment sessions and increase obliteration rates. A period of six months is expected to provide sufficient time for the tissue surrounding the irradiated AVM to heal any sublethal damage that may occur during radiosurgical sessions. Reducing the amount of radiation that reaches the surrounding normal brain tissue can also lower the risk of delayed white matter changes or cyst formation following VS-SRS for large AVMs [37]. Seymour et al. retrospectively compared the VS-SRS treatment outcomes for 63 AVMs larger than 10 mL during two eras: Era 1 (from 1992 to March 2004) and Era 2 (from May 2004 to 2008). In the Era 2 group, the target volume of each stage was reduced (median, 15.0 cc to 6.8 cc), the dose per stage was increased (median, 15.5 Gy to 17.0 Gy), and the interval between stages was shortened (median, 5.8 to 3.7 months) compared to the Era 1 group. The rate of near or complete obliteration was significantly higher in Era 2 than in Era 1 (21% vs. 68% at 5 years). Additionally, the near or complete obliteration rate was significantly higher for AVMs treated with a dose of at least 17 Gy per stage. The complication rates were 29% and 13% in Eras 1 and 2, respectively. The authors recommended that the irradiation volume of each stage should be ≤8 mL and that the marginal dose should be ≥17 Gy [62]. 

Two approaches were employed to strategize the planning of VS-SRS. The first involved the creation of a plan that covered the entire nidus during the initial stage. The plan was divided into 2–4 volume stages, serving as the first volume stage and pre-plans for the following stages. In the second method, each volume stage was planned solely on the date of each treatment. This method could potentially reduce the underdosing of the junction zones between the nidus subvolumes and accommodate any anatomical alterations that may be associated with a treatment response [18]. This approach enabled more precise target delineation and higher-quality dose plans, thus preventing dose overlap within and outside the nidus [83].

The planning strategies in VS-SRS are as follows:

- The time interval between sessions should be 3–6 months [37,84,85].
- The components are divided mainly in the vertical direction (z-axis direction) [85].
- The AVM treatment plan should start from the deepest region to the most superficial region, and from the medial to the lateral region [86].
- The nidus should be divided according to the territories of the contributing arteries [87].
- The components with main feeders should be irradiated first [85].
- The portion of the AVM associated with the major draining veins should be irradiated last in order to minimize the possibility of increasing the hemorrhage risk due to early venous outflow obstruction [37,85].
- It is imperative to make every possible effort to guarantee that the radiation overlap is limited to the AVMs and not in the normal brain tissue [37].
- The minimum marginal dose should be 17 Gy or greater, depending on the AVM location [62,64].
- The irradiation volume of each stage should be ≤8 mL [62].
- Additional isocenters with low weights should be added within the volume described by 17 Gy to increase the volume of AVM receiving ≥20 Gy [81].
- An irradiation volume of 18 Gy (V_18Gy_) < 10 mL should be maintained when possible [85].
- Smaller volumes per stage should be used for deep AVMs to decrease the incidence of symptomatic ARE [88].
- Low-dose SRS with repeat SRS could be an option for moderate-sized AVMs in a deep location [57].

The primary goal of AVM treatment is complete obliteration. However, complete obliteration of large AVMs remains challenging and takes longer after VS-SRS. Previous studies have reported that patients experience neurological improvements even without complete obliteration. The progressive decrease in the volume of large AVMs with VS-SRS might positively alter the hemodynamics within the nidus. This can potentially lead to clinical benefits such as a reduced risk of hemorrhage, improvement in seizure control, and the reversal of neurological deficits caused by chronic vascular steal [89,90,91]. 

### 5.3. DS-SRS vs. VS-SRS

Table 1 lists the published results of DS-SRS and VS-SRS for large AVMs. Two systematic reviews comparing VS-SRS with DS-SRS (including HSRT) identified higher rates of obliteration with the VS-SRS approach, with similar rates of toxicity favoring the VS-SRS approach [92,93]. 

Moosa et al. previously performed a systematic review of VS-SRS and DS-SRS for the treatment of large AVMs (>10 cm^3^). The mean complete obliteration rates in the DS and VS groups were 22.8% and 47.5%, respectively, while the mean rates of symptomatic radiation-induced changes in these groups were 13.5% and 13.6%, respectively. The mean rates of cumulative post-SRS latency hemorrhage in the DS and VS groups were 12.3% and 17.8%, respectively. The mean post-SRS mortality rates were 3.2% and 4.6% in the DS and VS groups, respectively. The authors proposed that VS-SRS provides higher rates of obliteration with comparable rates of complications to DS-SRS. Therefore, VS-SRS may be a more effective approach for treating large AVMs that have not been successfully treated using single-session SRS [93]. Ilyas et al. [92] also conducted a systematic review of 11 VS-SRS and 10 DS-SRS for large AVMs (>10 cm^3^), comparing 299 and 219 patients by adding new studies to the prior data by Moosa et al. [93], and reported that the mean complete obliteration rate was 41.2% for VS-SRS and 32.3% for DS-SRS. The mean rates of symptomatic RIC and post-SRS hemorrhage were 14.0% and 18.8%, respectively, for VS-SRS, and 12.5% and 11.6%, respectively, for DS-SRS. They suggested that, from the perspective of achieving complete obliteration, VS-SRS is more beneficial, but DS-SRS is associated with fewer adverse events [92]. Fogh et al. exported the target and normal tissue contours from gamma knife radiosurgery to CyberKnife SRT in seven pediatric AVM cases treated with VS-SRS, yielding the same level of target coverage and conformity indices. They further revealed a mean reduction of 18.7% ± 7.3% in biologically equivalent 12-Gy normal brain volume in VS-SRS. They concluded that VS-SRS is more beneficial than hypofractionation in providing a higher dose to the target, and achieving better protection of normal brain tissue in the treatment of large AVMs [94].

## 6. Factors Related to Treatment Outcomes

A recent matched cohort analysis including 149 patients in each cohort to evaluate the effect of AVM location on repeat SRS outcomes split the patients into the deep (brainstem, basal ganglia, thalamus, deep cerebellum, and corpus callosum) and superficial cohorts. The authors concluded that AVMs located in a deep region had significantly lower favorable outcomes and obliteration rates compared with superficial lesions after repeat SRS, although rates of latent period hemorrhage and RIC were not significantly different [95]. A meta-analysis including 2508 patients with deep-seated AVMs reported that the mean obliteration rates were 67% and 65% in brainstem and basal ganglia/thalamus, respectively. The mean incidence of hemorrhage was 7% for the brainstem and 9% for basal ganglia/thalamus AVMs. The authors concluded that SRS appears to be a safe and effective modality for treating deep-seated AVMs [96]. As previously mentioned, Flickinger et al. observed significantly more symptoms when imaging changes developed in the midbrain and brainstem compared to the cerebral cortical or cerebellar locations according to the significant post-radiosurgery injury expression (SPIE) score (the frontal lobe had the lowest risk with an SPIE score of 0, whereas the pons/midbrain had the highest risk with an SPIE score of 10) [48]. Hence, when treating deep AVMs, there is a specific concern regarding the occurrence of ARE. One prior study involving VS-SRS, including moderate-sized AVM in a deep location, reported an unfavorable outcome, suggesting that low-dose SRS with repeat SRS could yield similar results as VS-SRS for these patients [57].

Essibayi et al. pooled data from 22 studies with 3469 patients (1316 pediatric and. 2153 adult aged over 18 years) in the meta-analysis and reported that there was no significant difference in the obliteration rate between the pediatric (61%) and adult (67%) cohorts. The post-SRS hemorrhage rates (5% pediatric, 6% adult) and symptomatic RIC rates (10% in both cohorts) were similar [97]. A multi-center retrospective analysis of patients treated with a planned prospective VS-SRS reported that age was not a significant predictive factor for obliteration. However, improved overall survival was correlated with younger age at VS-SRS in the multivariate analysis [18].

Many previous studies have identified that the total AVM volume was inversely correlated with obliteration when treating large AVMs [18,62,66,83]. Nevertheless, some investigations ha e reported that there was no significant correlation between AVM volume (size) and obliteration [64,81,98]. Regarding the type of AVM (compact vs diffuse), compact nidus architecture was a significant predictive factor for obliteration in the multivariate analysis [18,62,83]. 

A retrospective analysis on 791 patients with AVMs treated with SRS in a single center revealed that a modified Pittsburgh radiosurgical AVM score ≥ 1.2, VRAS ≥ 3, and SM grade ≥ 3 showed significant inverse correlations with nidus obliteration [66]. Graffeo et al. reported a meta-analysis incorporating 9 studies of 1634 AVMs consisting of 431 SM Grade III (88%), 186 SM Grade IV (11%), and 11 SM Grade V lesions (1%). They observed total obliteration and hemorrhage rates of 72% and 7% for SM Grade III and 46% and 17% for SM Grade IV–V lesions, respectively. High-grade AVMs exhibit diversity and heterogeneity, making it challenging to predict their response to SRS. Although the median obliteration rate was less than 50% in SM Grade IV–V lesions, the authors recommended SRS as a primary treatment with personalized treatment planning strategies for high-grade AVMs, unless there are specific features that make surgical removal a better option [99].

## 7. Asymptomatic Large AVMs

Following the results from A Randomized Trial of Unruptured Brain AVMs (ARUBA) and the Scottish Audit of Intracranial Vascular Malformations prospective AVM cohort study, which reported an equal or greater risk of any intervention than the natural history of AVM if left untreated for unruptured asymptomatic AVM, the risk-benefit profile of treatment for this subset of patients has been the subject of significant debate [100,101]. 

The outcomes of treatment of high-grade AVMs have been found to be worse after interventions in comparative studies on conservative management and intervention [36,102]. For example, one multi-center retrospective analysis of 110 unruptured SM Grade IV–V AVMs reported a relatively low obliteration rate (28%), along with high rates of post-SRS hemorrhage (20%), symptomatic RIC (13%), and death (7%). The authors concluded that the risk-to-benefit profile of SRS for unruptured high-grade AVMs was poor and suggested that conservative management was superior to single-session SRS [98]. However, some studies have indicated that intervention may be beneficial for appropriately selected patients, as it could modify the natural history of these lesions with acceptable treatment-related morbidity rates [103,104,105]. A recent meta-analysis comprising 1620 ARUBA-eligible patients (36% asymptomatic) who underwent SRS identified SRS achieved obliteration to 68% with a post-SRS hemorrhage risk of less than 10%. The authors suggested that SRS has a positive risk–benefit ratio for ARUBA-eligible patients who are carefully chosen, especially those with smaller AVMs [106]. Individuals who have experienced a previous hemorrhage or are experiencing worsening neurological symptoms due to AVMs have a risk–benefit ratio that makes intervention more reasonable. In such cases, it is important to consider all possible treatment options. When managing asymptomatic large AVMs, which are believed to be more beneficial for treatment, more elaborate radiosurgical planning to minimize the risk of latent period hemorrhage and ARE and long-term follow-up are required to monitor delayed ration-induced toxicities.

## 8. Future Study

Four-dimensional (4D) flow MRI is a non-invasive technique used to evaluate time-resolved 3D blood flow velocities, providing complete coverage of the entire volume of the brain. 4D Flow MRI can further measure cerebral hemodynamics at the level of individual voxels over the entire volume. This method is distinct from structural imaging and provides unique information [107]. Prior research has demonstrated that 4D Flow MRI can be used to analyze intricate 3D blood flow patterns, identify the primary feeding arteries and draining veins, and measure changes in cerebral blood flow distribution following staged embolization treatment of AVM [108,109]. Large AVMs have a complex angioarchitecture, and may have multiple feeding vessels. In VS-SRS, as previously mentioned, it is recommended to first divide the nidus according to the territories of the contributing arteries [87], and to only then irradiate the components involving the main feeder [85]. 4D Flow MRI can facilitate not only radiosurgical planning in VS-SRS for large AVMs by identifying the discrete portion of the nidus that is fed by separate arterial supplies, but also the measurement of hemodynamic changes in AVMs after VS-SRS, even in the early phase, when volumetric changes of the nidus are not prominent in the structural images. Srinivas et al. further demonstrated that appreciable changes, including a decrease in arterial flow in the primary feeding artery and flow in the draining vein within the first 6 months after SRS, occurred earlier than the structural changes on standard MRI/MRA [110]. Flow mapping capabilities can be used to assess alterations in flow connectivity in complex AVMs with multiple feeding and draining vessels, and may aid in refining SRS treatment targets and capture treatment responses and changes in blood flow within AVMs during the latent period until nidus obliteration [111,112]. 

Until recently, the available medical treatments for AVMs have been limited. Some researchers have identified that the level of vascular endothelial growth factor (VEGF) is elevated in surgically resected specimen [113,114,115]. Walker et al. developed an AVM mouse model by focal *Alk1* gene deletion and human VEGF stimulation and revealed that VEGF antagonism by bevacizumab (Avastin) might reduce the number of dysplastic vessels [116]. Further investigation is needed to see if Avastin can be effectively used as a specific medical treatment for the human AVM, by inhibiting VEGF. Furthermore, Ferreira et al. specifically reported VEGF-D overexpression in AVM and identified that microRNA-18a reduced the VEGF-D level and AVM-derived brain endothelial cell proliferation [117]. Gene and cell therapy combined with SRS has clinical potential for novel and personalized therapeutic strategies for large intractable AVMs. 

## 9. Conclusions

VS-SRS appears to be a beneficial approach for the treatment of large AVMs at risk of obliteration failure. However, dose selection must balance the chances of obliteration and risk of ARE. The suggested time interval between VS-SRS sessions is 3–6 months. The nidus should be divided based on the territories of the contributing arteries. The components that get the main blood supply should be treated with radiation first, while the portion of the AVM associated with the major draining veins should be treated last. The minimum recommended marginal dose should be 17 Gy or higher, depending on the location of the AVM. To reduce the incidence of symptomatic ARE, it is advisable to utilize a smaller volume per stage for deep AVMs. It is also advisable to consider utilizing low-dose SRS in combination with repeated SRS as a potential treatment approach for AVMs of a modest size located in a deep region. An elaborate radiosurgical plan to avoid radiation overlap within the normal brain tissue and minimize the risk of latent period hemorrhage is therefore necessary, particularly when treating asymptomatic large diffuse AVMs. Further studies involving 4D flow MRI to facilitate radiosurgical planning and monitor treatment responses are expected to improve treatment outcomes for large AVMs.

## Figures and Tables

**Table 1:** Literature review of staged SRS for large AVMs.

Authors & Year & Study Design.	DS or VS	No. of Patients		Follow-Up	Median Volume (cm^3^)	Median Marginal Dose (Gy)	No. of Stages	Time between Stages (Months)	Obliteration		Hemorrhage	ARE
Karlsson et al., 2007 [74] MC, R	DS	89		NA	14 (9–56)	First: 15 (10–25) Second: 18 (15–25)	42 pts; 2, 2 pts: 3	At least 35	62%		7%	7%
Kim et al., 2010 [76] S, R	DS	44		109.4 months (27–202)	Mean 48.8 (30.3–109.5)	Mean 13.9 (8.4–17.5)	23 pts: 2 8 pts: 3 2 pts: 4	At least 26	34.1%		6.8%	4.5%
Seymour et al., 2016 [62] S, R	VS	Era 1 (1999–2004)	33	8.6 years	27.3 (13.5–68)	15.5 (12–18)	2 stages + salvage SRS	Median 5.8 (3.1–31.1)	Near or complete 3 yr-5%, 5 yr-21%		11 pts experience 15 hemorrhages	29% persistent-16%
		Era2 (2004–2008)	30	4.6 years	18.9 (8.6–65.9)	17 (16–18)		Median 3.7 (2.0–6.7)	Near or complete 3 yr-23%, 5 yr-68%		7 pts experience 8 hemorrhages	13% persistent-3%
Seymour et al., 2020 [18] MC, R	VS	257		5.79 years	23.25 (7.7–94.4)	17 (12–20)	224 pts: 2 26 pts: 3 7 pts: 4	3–6	<17 Gy	5 yr-6.8%	3.7% per year total 46 hemorrhages	28.7% permanent-9.8%
									≥17 Gy	5 yr-26.7%		
Kano et al., 2012 [64] S, P	VS	47		87 months (0.4–209)	22.0 (10.2–56.9)	16 (13–18)	2–4 16 pts: additional SRS	Median 4.9 (2.8–13.8)	3 yr-7%, 4 yr-20%, 5 yr-28%, 10 yr-36%		1 yr-4.3%, 2 yr-8.6%, 5 yr-13.5%, 10 yr-36%	13%
Franzin et. al., 2016 [65] S, P	VS	20		45 months (19–87)	15.9 (10.1–34.3)	20 (18–25)	2	Mean 15 ± 9	42%		10%	5%
Kano et al., 2018 [81] S, R	VS	60		82 months (0.4–206)	First: 11.6 (4.3–26), Second: 10.6 (2.8–33.7)	16 (13–18)	2	Median 4.5 (2.8–13.8)	3 yr-4%, 4 yr-13%, 5 yr-23%, 10 yr-27% When ≥17 Gy and 20 Gy volume ≥ 63% 5 yr-61%, 10 yr-70%		1 yr-1.7%, 2 yr-5.2%, 3 yr-7.0%, 5 yr-9.0%, 10 yr-25.2%	8.3%

ARE, adverse radiation effect, DS, dose-staged; MC, multicenter; P, prospective; pts, patients; R, retrospective; S, single; VS, volume-staged; yr, years.

## Data Availability

Not applicable.
